# Data Analytics and Administrative Decision-Making in Nursing Management: A Systematic Review

**DOI:** 10.1155/jonm/4344147

**Published:** 2025-11-05

**Authors:** Nathidathip Darach, Min Su Kim, Wasinee Wisesrith, Eileen G. Collins

**Affiliations:** ^1^Faculty of Nursing, Chulalongkorn University, Bangkok, Thailand; ^2^College of Nursing, University of Illinois Chicago, Chicago, Illinois, USA

**Keywords:** data analytics, decision-making, evidence-informed decision-making, nurse managers, nursing informatics, nursing management

## Abstract

**Aim:**

This systematic review aimed to investigate the impact of data analytics on nurse managers' administrative decision-making process and roles.

**Background:**

The growing integration of data analytics in health care has accelerated the shift toward data-driven decision-making in nursing management, aiming to optimize patient care quality and enhance organizational performance within digital healthcare environments. Nurse managers play a pivotal role in leveraging data analytics to support evidence-based management, facilitating more informed, efficient, and strategic administrative decision-making.

**Method:**

This systematic review was conducted in accordance with PRISMA guidelines. A comprehensive search strategy was employed to identify relevant studies published from 2019 through 2024 using four electronic databases—PubMed, CINAHL, MEDLINE, and Embase. A total of 2051 studies were screened, and 83 studies were eligible for full-text screening according to the established inclusion and exclusion criteria. Eight different quality assessment tools were applied. Data tabulation and narrative synthesis were employed.

**Results:**

Twenty-one studies representing eight different study designs were included in the review. There were diverse applications of data analytics across four analytics levels: descriptive (*n* = 4), diagnostic (*n* = 2), predictive (*n* = 9), and prescriptive (*n* = 1). Additionally, integrated approaches combining two levels of analytics were identified (*n* = 5).

**Conclusion:**

The integration of data analytics into nursing management has the potential to enhance an administrative decision-making process across diverse nursing management roles, particularly in four key areas: improving patient care quality, strategic management, nurse staffing and work engagement, and nursing management during health crises.

**Implications for Nursing Management:**

Strengthening nurse managers' analytical and digital competencies through targeted education and continuous training is essential. Ensuring supportive infrastructure can enable more informed, efficient, and evidence-based management, ultimately leading to improved healthcare quality and operational performance. Future research should explore the long-term impact and broader applicability across diverse healthcare settings.

## 1. Introduction

The digital revolution has ushered in a new era of digital healthcare, characterized by the exploitation of the great potential of digital technologies to optimize patient care quality and improve healthcare organizational management since the early twenty-first century [1]. In this context, the health information system (HIS), a core component of digital health care, serves as a critical infrastructure in the modern healthcare ecosystem [2–4]. Data generated through HIS provide substantial value and a wealth of information in the form of big data for healthcare systems [5].

Recognizing its importance, the World Health Organization (WHO) framework recommends that HIS should contribute to producing intelligence outputs that provide information and analysis for decision-making. In turn, this can empower healthcare providers and policymakers to make informed, evidence-based decisions while considering both the potential limitations and benefits of HIS [4].

The COVID-19 pandemic has further accelerated the global expansion of digital advances in healthcare delivery [6]. Despite this evolving landscape, traditional analytical approaches remain insufficient to manage the vast volume and complexity of healthcare data, particularly in delivering essential information to support healthcare services as well as decision-making among providers. Consequently, the emergence of health data science—focused on the development and application of data analytics—is driving significant advances across the healthcare sector [7]. Integrating healthcare data to leverage data analytics, which involves systematically applying statistical analysis, big data, and computational techniques to extract actionable insights, aims at informing decision-making processes to enhance care quality and organizational performance [8]. Moreover, ongoing studies in the use of data analytics in healthcare settings have underscored its potential and precision in delivering the best available evidence—both internal (organizational) and external (scientific). These interconnected sources of evidence are critical for supporting evidence-informed decision-making in nursing management [9, 10].

Administrative decision-making (ADM) by nurse managers refers to a complex cognitive process involving rational and critical thinking, the use of the best available evidence, and clinical and managerial expertise to achieve optimal decisions for managerial purposes. This type of decision-making profoundly influences daily healthcare delivery. Effective decision-making requires application of effective strategies to achieve the best solutions to healthcare problems [11]. To conceptualize this decision-making process, Chisengantambu-Winters et al. [12] developed the decision-making dependency (DMD) model, which emphasizes contextual and personal factors influencing nurse managers' decisions; however, their model does not explicitly incorporate data- and information-driven processes. This omission constrains its relevance in the digital era, where electronic health records, analytics, and decision-support systems (DSS) are integral to evidence-based, technology-enabled decision-making. While Watson's [13] nurse leader decision-making within complex adaptive system model reflects the realities of today's dynamic healthcare environment—nonlinear, adaptive, and context-sensitive, with strategies shifting according to whether a situation is simple, complicated, complex, or chaotic—it provides limited methodological structure for empirical study. For these reasons, we chose to use Oetjen et al.'s [14] ADM model as a conceptual framework for our review. Although Oetjen et al.'s model was established nearly 2 decades ago, it highlights the role of information in guiding nurse managers' decision-making, an alignment that is inherent to data-driven healthcare systems. In this context, insights derived from data analytics have emerged as a valuable foundation for evidence-informed decision-making in nursing management. The ADM model includes a six-step process consisting of defining the problem, developing and ranking relevant criteria, collecting information, formulating and ranking solutions, jointly choosing the best solution(s) with the healthcare team, and enacting and monitoring the chosen solution(s). Especially given the transition to digital health care, this framework provides valuable insights into means of enhancing and simplifying the ADM process of nurse managers, particularly in maintaining a balance between human judgment and technological advances.

In the advancement of modern healthcare systems, nurse managers face critical challenges in integrating digital technologies to make timely and effective administrative decisions that encompass their wide range of roles and responsibilities [15, 16]. However, the integration of data analytics into nurse managers' ADM process presents both substantial challenges and transformative opportunities for addressing complex healthcare delivery problems [17]. By utilizing data analytics, this innovative practice can empower nurse managers to make informed decisions, supporting their multifaceted roles and enabling them to navigate healthcare complexities more effectively [16]. Moreover, these tools offer valuable support for decision-makers with limited management experience [18]. For nurse managers to utilize data analytics tools for targeted purposes, Stoumpos et al. [19] pointed out that appropriate analytical approaches need to be applied to deliver accurate and useful results. Therefore, a foundational understanding of various types of analytics and their limitations is essential to assess their implications effectively. However, the extent to which nurse managers are utilizing data analytics in nursing management and its impact on their decision-making processes remains underexplored.

Since 2019, researchers have shifted their focus from digital transformation and its applications in health care to its effective implementation [6, 19, 20]. Despite this trend, no review study has comprehensively examined the use of data analytics to support nursing management roles. A study by Moorhead [21] proposed 10 paths for nurse managers to implement data-driven care using the Nursing Outcomes Classification (NOC) and Nursing Interventions Classification (NIC) frameworks. These paths included, for example, identifying frequent patient problems, nursing interventions, and outcomes, use of clinical decision models that support clinical reasoning, and use of nursing data to determine staffing needs based on NIC or NOC [22]. While these guidelines provide valuable insights for improving patient-centered care and nursing interventions, their applicability may be limited in addressing the broader administrative and operational decision-making responsibilities of nurse managers, which extend beyond clinical needs alone. Similarly, Wieben et al. [23] presented data science applications with a focus on nursing practice indicators, and Clancy [24] evaluated various types of digital tools and technologies for nurse managers' use in improving nurse productivity. However, the limitations of these frameworks highlight the need for more robust approaches and customizable data solutions to enhance nurse managers' capacity to make optimal decisions across their management roles.

In this review, we systematically identified, appraised, and synthesized existing evidence to examine how nurse managers use data analytics tools and techniques in their ADM practices. Understanding the potential benefits and challenges of analytics, evidence-informed decision-making in nursing management can lead to improved decision-making processes and ultimately enhance the quality and efficiency of healthcare delivery.

## 2. Methods

### 2.1. Design

This systematic review was conducted and reported in accordance with the Preferred Reporting Items for Systematic Reviews and Meta-Analyses (PRISMA) 2020 guideline for systematic reviews [25].

### 2.2. Ethical Considerations

Ethical approval of this research was not required as data were retrieved and synthesized from previously published studies.

### 2.3. Search Strategies

A comprehensive search strategy was employed to identify relevant studies published from January 1, 2019, through December 15, 2024, in four electronic databases: PubMed, CINAHL, MEDLINE, and Embase. Manual searches of the reference lists of included journal articles were also conducted to identify additional relevant articles. The search strategy was structured using the Population, Intervention, Comparison, and Outcomes (PICO) framework. For example, PubMed's Medical Subject Headings (MeSH)—including “Nurse Administrators,” “Data Science,” and “Decision Making”—as well as supplementary keywords, such as “digital analytics,” were combined using Boolean operators (“AND,” “OR”) to optimize retrieval. To improve the search's sensitivity, keywords from the included studies were also used in the search process. All search terms and keywords were reviewed and confirmed by a health science librarian with expertise in search strategies.

### 2.4. Selection Criteria and Retrieval Strategies

Inclusion and exclusion criteria were applied to guide the selection of studies. These criteria were selected to ensure that the review captured a broad range of data analytics utilized for nursing management. Studies were included if they (1) impacted nurse managers' decision-making, (2) examined use of a data analytics at any level—potentially ranging from descriptive and diagnostic to predictive and prescriptive analytics—and related factors enhancing or associated with nursing management roles, (3) involved primary research or quality improvement project, (4) reported quantitative results, and (5) were peer-reviewed journal articles published in English. We elected to accept the definition of data analytics and advanced analytics implied or stated in each eligible study, and these definitions encompassed a variety of digital analytics tools, practices, and interventions used to support decision-making in healthcare settings. While this approach enabled us to capture the full scope of conceptualizations currently employed, it also introduced variability that should be considered when interpreting the findings.

Qualitative studies and gray literature were excluded, as were studies in which the primary population was not nurses or the outcome of interest was not associated with the utilization of data analytics for decision-making. Additionally, given the rapid advances in digital technologies and their exponential implementation in health care since 2019 (Dionisio et al., 2023; [19, 20]), only recent studies—those published in the past five years—were included to ensure that the evidence reported was up to date. Consequently, studies published before 2019 were excluded.

**Figure 1:**
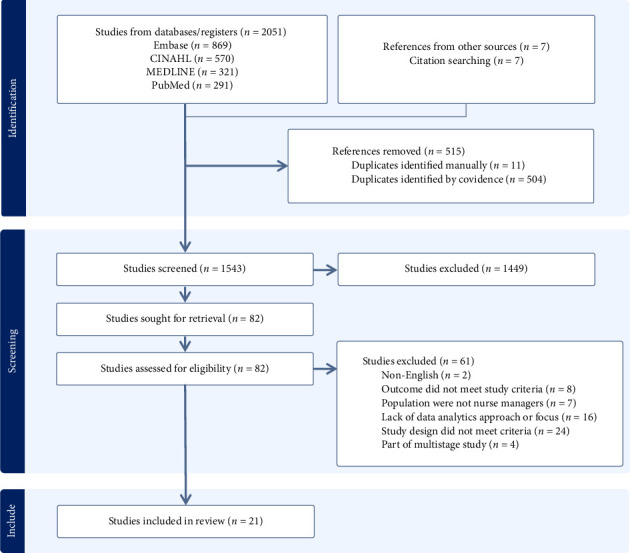
PRISMA flow diagram.

A total of 2051 studies were screened, the titles, abstracts, and search terms, according to the eligibility criteria, and 83 studies were eligible for full-text screening. Next, the full-text versions of potentially eligible articles were independently examined by two reviewers to exclude those that did not meet the eligibility criteria. The two reviewers discussed differences of opinion about study eligibility until a consensus was reached on the studies to be included in the review. The study selection process is summarized in the PRISMA flowchart in Figure 1.

### 2.5. Data Extraction and Synthesis

To maximize the accuracy of data extraction and synthesis, standardized strategies were applied, including establishing a structured data extraction form, extraction and verification of data by two independent reviewers (N.D. and M.S.K.), statistical assessment of heterogeneity, and use of Covidence data extraction software. An Excel data-charting table was used to extract data from the full texts selected for inclusion. The data extracted from each study included the author(s) and year, research aim, study design, setting and sample information, characteristic and level of data analytics used, impact on nurse managers' decision-making, and quality assessment scores. As an additional check to ensure study rigor, the eligibility criteria were reapplied to all included full-text articles during data extraction. The results of the included studies were synthesized using narrative synthesis, a process involving summarizing and explaining information in words; during data synthesis, tables were used to compare the study characteristics and the extracted data.

### 2.6. Quality Appraisal

The quality of the included studies was appraised using quality assessment tools specific to particular study designs. Joanna Briggs Institute (JBI) critical appraisal tools [26, 27] were applied for analytical cross-sectional, case–control, cohort, and randomized controlled trial (RCT) studies. In addition, the Strengthening the Reporting of Observational Studies in Epidemiology (STROBE) tool [28], guideline for good evaluation practice in health informatics (GEP-HI) [29], risk-of-bias tool for prediction models (PROBAST) [30], and the Quality Improvement Minimum Quality Criteria Set (QI-MQCS) [31] were applied for other studies. Two reviewers independently appraised each study using the tools' scoring and grading criteria (high quality/excellent/good practice, moderate quality/good, poor/fair, and very poor/unsatisfactory). Discrepancies between the two reviewers' scores were resolved through discussion and, if necessary, in consultation with a third reviewer.

## 3. Results

### 3.1. Study Characteristics

Twenty-one studies were included (Table 1). They were conducted in the United States (*n* = 10), South Korea (*n* = 4), China (*n* = 2), Qatar (*n* = 1), Canada (*n* = 1), Australia (*n* = 1), the Netherlands (*n* = 1), and Jordan (*n* = 1). The study settings included tertiary hospitals (*n* = 7), general hospitals (*n* = 5), specialized or university-affiliated hospitals (*n* = 4), Veterans Administration (VA) hospitals (*n* = 3), and an outpatient clinic (*n* = 1). In one study, participants were drawn from a national survey dataset.

Eight different study designs were represented. They included cross-sectional studies (*n* = 4), case–control studies (*n* = 2), an RCT (*n* = 1), cohort studies (*n* = 2), a prediction model study (*n* = 1), observational studies (*n* = 3), health informatics studies (*n* = 5), and quality improvement projects (*n* = 3).

The quality of the studies was appraised using eight different tools as shown in Table 2. In total, 19 studies were appraised as showing high-quality/excellent/good practice/met quality criteria, while two studies were found to be of moderate quality and posing risk of bias.

### 3.2. Data Analytics Characteristics

In health care, data analytics refers to the systematic application of statistical and computational methodologies to large and complex datasets, with the objective of deriving meaningful insights, identifying patterns, and generating actionable knowledge that informs clinical and ADM, improves patient outcomes, and enhances organizational performance [8].

Eligible studies were included based on their eligibilities. Specifically, we considered the level of data analytics described as an important criterion, recognizing that these levels represent different stages of data analytics maturity and application in practice.

As shown in Table 1, 21 studies displayed diverse characteristics and levels of data analytics intended to enhance ADM of nurse managers. Studies reflecting the intersection of data science principles and practical decision-making needs in nursing management were categorized into four levels of data analytics based on practical guidance for nurses and healthcare sectors [7, 53]: descriptive (*n* = 4), diagnostic (*n* = 2), predictive (*n* = 9), and prescriptive analytics (*n* = 1). In addition, we included a fifth category—integrated data analytics—that was addressed in some studies (*n* = 5).

#### 3.2.1. Descriptive Analytics—Use of Data to Provide Information in Real Time

Four studies focusing on descriptive analytics aimed to enhance decision-making by providing real-time information and insights. Almagharbeh [32] investigated the use of AI-based DSS in critical care reporting improvements in patient care workflow with regard to time management and clinical decision-making. Baldwin et al. [33] assessed the coworker observation system (CORS), promoting a safe working environment. King et al. [45] demonstrated the use of real-time workforce data to proactively identify and mitigate nurse burnout. Moon [49] evaluated the nursing resources management information system (NRMIS), a resource allocation tool in a university hospital. This electronic reporting tool offered objective data and useful insights for flexible nurse resource allocation.

#### 3.2.2. Diagnostic Analytics—Use of Data Collected From HIS to Identify and Generate Particular Information Patterns

Two studies examined methods to enhance decision-making transparency and operational insight in health care. Berkhout et al. [34] developed an algorithm using real-life datasets and synthetic data in an emergency unit to simulate nurse decision-making through visualization of decision logs. Duffield et al. [36] examined the effects of bed control adjustments and patient mix on nurses' workload within a tertiary hospital. Analysis of the ward-level administrative data was beneficial for informing staffing decisions and workload management. The methods examined in the two studies had common limitations, including dependence on specific data sources or developer expertise, that could affect their broader applicability.

#### 3.2.3. Predictive Analytics—Integration of Historical Data Patterns to Predict Outcomes and Identify Initial Actions

Nine studies on predictive analytics offered insights into workload management, environmental factors, and risk prediction in nursing. For example, Campbell et al. [35] examined big data from electronic medication administration records in a U.S. hospital, identifying workload factors affecting near-miss medication errors to support personalized interventions for risk reduction. Guo et al. [38] used four machine learning algorithms to develop a decision tree model—a visual and logical model used in machine learning and decision-making—for job crafting analysis and nurse burnout prediction in Chinese hospitals, and Havaei et al. [40] employed machine learning methods to identify key work environment predictors of nurse mental health in Canada. Howard [41] investigated allostatic load metrics in a VA hospital to guide nurse staffing decisions. The study utilized signal detection of allostatic load derived from EMR data, which was generated based on a Troubled Outcome Risk (TOR) scale. As a final example, [42] developed a machine learning tool for pressure injury (PI) risk in South Korea.

#### 3.2.4. Prescriptive Analytics—The Most Advanced Analytics Level, Use of Data to Generate Most Possible Outcomes and to Recommend Actions and Strategies Based on Predictions

One study involving prescriptive analytics focused on optimizing healthcare resources and decision-making processes. Ghayoomi et al. [37] utilized mathematical programming and queueing models to estimate maximum hospital capacity for COVID-19 cases in a 200-bed urban hospital model, providing tools for enhanced resource allocation.

#### 3.2.5. Integrated Data Analytics—Integration of Two of the Analytics Functions

Among the five studies that integrated two levels of data analytics, Hadid et al.'s [39] utilized simulation-based optimization and clustering techniques to enhance scheduling efficiency at an outpatient chemotherapy center. This combination of predictive and prescriptive analytics reduced patient wait times and streamlined appointment management, showing significant improvements in operational efficiency. Kang and Kim [43] conducted a RCT to evaluate a clinical DSS (CDSS) for postembolization pain management across multiple hospitals, demonstrating its effectiveness in standardizing treatment approaches. Their predictive analytics provided risk prediction, while prescriptive analytics generated standardized care recommendations. Three studies integrating diagnostic and predictive analytics focused on data-driven strategies to improve healthcare management. Kohn et al. [46] assessed ward capacity data across multiple hospitals, providing predictive insights that supported resource allocation. Lindberg et al. [47] focused on identifying key factors in fall prevention, supporting proactive patient safety measures. Yan et al. [52] constructed a machine learning model to detect high-risk patients, offering predictive capabilities for nurse managers. However, their model's reliance on intraoperative data appeared to restrict its preoperative applications.

Overall, 21 studies reviewed demonstrated that data analytics have the potential to revolutionize ADM on the part of nurse managers. However, the reviewed studies shared several limitations, such as data retrieval from limited sources, restricted generalizability due to specific organization contexts, reliance on historical data, the use of unmeasured confounders that introduced potential biases affecting predictive accuracy, and overdependencies on experts or interdisciplinary health informatics teams.

## 4. Discussion

Data analytics in healthcare involves the systematic use of statistical and computational methods to transform data into actionable insights that inform decisions, improve patient outcomes, and enhance organizational performance [8]. In this systematic review, we aimed to gather the most recent evidence on nurse managers' utilization of data analytics and its impact on their ADM. Our inclusion of studies across varying levels of data analytics maturity was intentional, as it allowed us to reflect the full continuum of data analytic applications in nursing management. This heterogeneity highlights both the opportunities and challenges inherent in advancing from descriptive or diagnostic analytics toward more sophisticated predictive and prescriptive approaches that carry greater potential for supporting evidence-based decision-making.

To provide specific insights, the associations observed between data analytics and nursing management roles are discussed across four key dimensions below [16, 54–57]. Additionally, the impacts of data analytics—descriptive, diagnostic, predictive, prescriptive, and integrated—on these four dimensions of nursing management roles are presented in Table 3. Furthermore, the findings reveal the essential role of data analytics in supporting Oetjen et al.'s [14] the six-step ADM process among nurse managers, as illustrated in Table 4.

### 4.1. Improving Patient Care Quality

Several studies highlighted the impact of data analytics in enhancing patient care quality by providing data-driven insights into nurses' clinical performance. Nurse managers' administrative decisions—shaped by operational structures and resource allocation—play a critical role in creating an environment that supports effective clinical decision-making. The use of advanced monitoring and diagnostic tools enabled nurse managers to identify inefficiencies, streamline clinical workflows, and implement evidence-based interventions that directly improved patient outcomes. These tools also contributed to optimizing care protocols and promoted greater adherence to best clinical practices among nurses, ultimately elevating the overall standard of patient care.

### 4.2. Strategic Management

Data analytics was shown to be instrumental in strategic planning and long-term management within healthcare organizations. Big data integration facilitated the analysis of patterns and trends across large datasets, informing high-level decisions, such as budget allocation for nursing workforce management, policy development, and organizational performance tracking. On the whole, the strategic use of data analytics enabled proactive management and provided an evidence-informed approach to achieving organizational goals, especially in complex healthcare environments.

### 4.3. Nurse Staffing and Work Engagement

Descriptive, predictive, and prescriptive analytics were particularly valuable in managing nursing human resources as well as promoting healthy work environments. Tools that forecast patient admissions, staffing needs, role-related stressors, and nurse turnover empowered nurse managers to optimize workforce planning and guided them in devising strategies to improve staff engagement. Evidence-informed decision-making of nurse managers facilitated more precise deployment of nursing staff, balanced workloads, and improved scheduling, all of which are crucial in maintaining a supportive work environment and reducing staff burnout.

### 4.4. Nursing Management During Health Crises

The utility of digital analytics became especially apparent during health crises. During these events, having real-time data was critical for rapid response and resource management. Some studies highlighted use of analytics to monitor patient surges, allocate resources efficiently, and manage critical situations with agility. In crisis scenarios, nurse managers could leverage predictive models to anticipate challenges as well as prescriptive tools to streamline emergency protocols, enhancing the healthcare system's resilience and responsiveness.

This synthesis highlights recent findings across four key administrative areas where data analytics played a crucial role in enhancing nurse managers' decision-making. In the realm of patient care quality, nurse managers were equipped with evidence-based insights—both internal and external evidence—into nurses' clinical performance, allowing efficient interventions and enhancing care standards. As for strategic management, big data informed high-level decisions on budgeting, workforce planning, and policy formulation, creating a data-driven, proactive approach within healthcare organizations. In addition, nurse staffing and work engagement were optimized by using predictive analytics that assisted in workforce planning, workload balancing, and retention efforts, contributing to a supportive work environment. Finally, with respect to analytics-informed nursing management during health crises, use of real-time data supported prior simulation of emergency scenarios as well as agile responses and resource allocation, boosting overall system resilience.

Across the studies reviewed, data analytics demonstrated significant potential to enhance ADM among nurse managers. However, several barriers to implementing data analytics in diverse healthcare settings were consistently observed. These included the reliance on fragmented or limited data sources that can restrict data quality and generalizability of findings bound to specific organizational contexts. In addition, the presence of unmeasured confounders raised concerns regarding predictive validity. Moreover, the dependence on expert consultants or interdisciplinary informatics teams highlighted ongoing challenges in building sustainable, locally embedded analytic capacity within nursing management. To address these challenges, the need for end users' engagement through targeted training on digital literacy and competency development was also emphasized as essential to realizing the full potential of implementing data analytics in nursing administration practices.

## 5. Limitations and Strengths

Limitations are inherent to both the studies included and the review itself. First, the generalizability of findings is constrained by the predominant reliance on data from specific organizational contexts, as most studies focused on single-site settings or narrow populations. Additionally, the exclusion of publications in languages other than English, qualitative studies, gray literature, and articles published as part of mutistage studies may have inadvertently omitted insights regarding the experiential and contextual nuances of data analytics. Furthermore, excluding studies published before 2019 poses potential limitations, although rapid technological advancements may make earlier findings less applicable. Finally, uncertainties and variations regarding the definition and scope of data analytics tools across studies introduce challenges in drawing uniform conclusions. For example, some researchers offered no clear definition of the data analytics employed in their studies, forcing us to infer their nature as well as their level. In addition, some studies focused on the application of data analytics to improve patients' clinical outcomes; in such cases, we had to evaluate the relationship of such outcomes to nurse manager's decision-making.

However, this review has significant strengths that deserve mention. First, we include various study designs—ranging from cross-sectional studies and RCTs to health informatics applications—that provide a comprehensive understanding of how data analytics tools are employed across different healthcare settings. This diversity enhances the robustness of the review by capturing multiple perspectives on the impact of data analytics on nursing management. Other study strengths include its adherence to PRISMA guidelines and use of different quality appraisal tools tailored to particular study designs, both ensuring that the review's findings are grounded in rigorous evaluation.

## 6. Conclusion

This systematic review highlights the integral role of data analytics in supporting nurse managers' ADM by providing actionable insights across diverse nursing management roles. It encompasses diverse applications of data analytics across descriptive, diagnostic, predictive, and prescriptive analytics. Moreover, it demonstrates that use of data analytics holds considerable promise for improving decision-making to enhance patient care quality, as it can be applied to support strategic management, optimize staffing and work engagement, and guide nursing management during health crises. A number of significant challenges also emerged across the included studies, particularly variability in the analytic tools employed and limited organizational capacity to integrate advanced analytics into decision-making. Although such limitations are apparent in the studies reviewed, the potential benefits of integrating data analytics into nursing administration are also clearly visible.

## 7. Implications

The findings from this review underscore the growing role of data analytics in data-driven health care and have profound implications for nursing management, education, and policy. Data analytics enable nurse managers to optimize their ADM. Given the need for digital competencies, integration of targeted education and training into nursing curricula and continuous professional development for nurse managers are crucial to bridging digital literacy gaps and ensuring that nurse managers are equipped to effectively leverage analytics tools. At the policy level, policymakers should prioritize investments in analytics infrastructure and support systems to ensure interoperability, accessibility, user-centric design, and seamless integration into existing infrastructures and workflows. Future research should explore the longitudinal impact and broader applicability of data analytics across diverse settings, including resource-constrained environments, and should address technical and other obstacles to these tools' legitimacy. Ultimately, we believe that data analytics merits widespread adoption to enhance healthcare systems in settings with both abundant resources and resource limitations globally.

## Figures and Tables

**Table 1:** Data extracted from the included studies.

Study no.	Author(s) and year	Aim of study	Study design	Setting and sample information	Characteristic of the tool and level of data analytics addressed	Impact on nurse managers' decision-making	Quality assessment scores
1	Almagharbeh [32]	To examine the effects of AI-based decision support systems (DSS) on nursing workflows in critical care units (CCUs).	Cross-sectional study	Critical care units, and two government and three private hospitals in Amman, Jordan. The sample consisted of 112 nurses.	Characteristic: mobile health applications, wearable sensors, electronic health records (EHR)–integrated alerts, and clinical practice guidelines as AI-based DSS tools. Level: descriptive analytics	Improved workflow management by enhancing nurses' clinical decision-making.	High-quality (JBI critical appraisal checklist for analytical cross-sectional studies)

2	Baldwin et al. [33]	To assess the feasibility and monitor the fidelity of implementing the coworker observation system (CORS) for nurse professionals.	A mixed-methods observational study	Keck Medicine of the University of Southern California, University of Iowa Health Care, and Vanderbilt University Medical Center Adult Hospital.	Characteristic: the coworker observation system (CORS) electronic reporting system. Level: descriptive analytics	Provided a transparent peer feedback tool and process for nursing staff to promote a safe-working environment in healthcare organizations.	Excellent (STROBE tool)

3	Berkhout et al. [34]	To develop an algorithm using decision logs to improve healthcare decision-making processes.	Design science research methodology (DSRM)	Real-life dataset simulation with synthetic data in emergency unit.	Characteristic: fuzzy classifier algorithm for decision discovery and visualization, Decision Model and Notation (DMN). Level: diagnostic analytics	Improved quality of care by enhancing transparency and efficiency of nurses' decision-making in emergency unit through visualization of decision logs.	Good practice (GEP-HI)

4	Campbell et al. [35]	To explore the use of big data in identifying workload factors affecting near misses in nursing.	Cross-sectional study	Medical–surgical unit in a midsize U.S. hospital, sample of 23 nurses, and 389 patients over a 60-day period.	Characteristic: analysis of big data from barcode medication administration (BCMA), electronic health records. Level: predictive analytics	Provided insights into workload factors impacting medication errors and supported personalized interventions for risk reduction.	High-quality (JBI critical appraisal checklist for analytical cross-sectional studies)

5	Duffield et al. [36]	To investigate the impact of changes in bed configuration and patient mix on nurses' workload in a specific hospital ward.	Multimethod case study	Twenty-eight-bed ward in a tertiary referral hospital, Queensland, Australia; approximately 3000 patient records analyzed.	Characteristic: Ward-Level Administrative Data Analysis: bed occupancy, transfers, length of stay, nurse staffing levels, skill mix, and analysis of major diagnostic categories (MDC). Level: diagnostic analytics	Highlighted the need for nurse managers to consider ward-level data and provided insights to inform staffing decisions and workload management.	Good practice (GEP-HI)

6	Ghayoomi et al. [37]	To estimate maximum hospital capacity for COVID-19 patient care under surge conditions using queueing theory and microsimulation	Mixed Methods: mathematical programming with queueing theory and Jackson networks, validated against a discrete-event simulation model	Generic 200-bed urban tertiary hospital model. Focused on ICU and isolation units during COVID-19 surges.	Characteristic: queueing theory and Jackson networks, microsimulation for modeling hospital capacity under surge. Level: prescriptive analytics	Provided tools for optimizing resource allocation, specifically for beds and staffing, to prepare for pandemic surges; informed strategic and operational decisions.	Good practice (GEP-HI)

7	Guo et al. [38]	To explore the impact of job crafting and leisure crafting on nurse burnout using machine learning models	Cross-sectional study	A total of 1235 nurses at four Chinese tertiary hospitals	Characteristic: Four Machine Learning Algorithms: logistic regression model, support vector machine (SVM), random forest, and gradient boosting tree. Level: predictive analytics	The tree model identified job crafting as the strongest predictor of burnout, suggesting that data-informed insights can guide nurse managers in developing targeted managements.	High-quality (JBI critical appraisal checklist for analytical cross-sectional studies)

8	Hadid et al. [39]	To improve outpatient chemotherapy appointment planning and scheduling through clustering and stochastic simulation optimization	Simulation-based optimization using clustering algorithms and stochastic models	Outpatient chemotherapy center in the Gulf region, real-world data from 246 patient appointments over 5 days	Characteristic: stochastic discrete simulation-based multiobjective optimization (SDSMO) linked with clustering algorithms (K-means, hierarchical, and self-organizing maps). Level: predictive analytics and prescriptive analytics	Enhanced scheduling efficiency, reduced waiting times, and balanced workload among nursing staffs.	Good practice (GEP-HI)

9	Havaei et al. [40]	To identify key work environment predictors of nurse mental health using machine learning methods	Cross-sectional study	A total of 4029 nurses in British Columbia, Canada	Characteristic: machine learning techniques, specifically random forest regression, to assess the relative importance of workplace factors. Level: predictive analytics	Provided evidence to prioritize work environment factors (e.g., workload management and psychological protection) for targeted managements.	High-quality (JBI critical appraisal checklist for analytical cross-sectional studies)

10	Howard [41]	1. To calculate allostatic load in hospitalized patients using big data from the electronic medical record (EMR) 2. To reduce length of stay (LOS) and patient adverse outcomes 3. To increase nurse scheduling flexibility in a mixed medical–surgical unit	Retrospective and prospective observational study	VA hospital's 15-bed medical–surgical unit; retrospective data from 2461 patients and prospective data from 117 patients	Characteristic: signal detection of allostatic load using the Troubled Outcome Risk (TOR) scale derived from EMR data; big data analytics. Level: predictive analytics	Helped manage nurse staffing, improved nurse staffing patterns to patient-need based, and reduced allostatic load as measured by the TOR scale.	Excellent (STROBE tool)

11	Lee et al. [42]	To develop a machine learning–based pressure injury (PI) prediction model and integrate it into clinical practice	Retrospective case–control study	Urban tertiary hospital in Korea; 1,389,660 general ward cases and 139,897 ICU cases	Characteristic: gradient boosting algorithm, logistic regression, and decision trees; integration into the electronic health record (EHR) as a clinical decision support system Level: predictive analytics	Provided real-time, evidence-based PI risk assessment, helped nurse managers allocate resources effectively and prioritize high-risk patients.	High-quality (JBI critical appraisal checklist for case–control studies)

12	Kang and Kim [43]	To evaluate the effectiveness of a clinical decision support system (CDSS) for managing postembolization syndrome (PES) after transarterial chemoembolization (TACE)	Randomized controlled trial	Four tertiary hospitals and one secondary hospital in two metropolitan areas; 40 nurses and 51 patients	Characteristic: machine learning–based CDSS, including random forest and logistic regression models, features included risk prediction and care recommendations. Level: predictive analytics and prescriptive analytics	Improved Care Quality: guided clinical decisions with tailored suggestions based on evidence-based practice guideline, promoted patient-centered care, and enabled proactive planning and resource allocation	High-quality (JBI critical appraisal checklist for RCT studies)

13	Kim et al. [44]	To develop a nurse turnover prediction model using machine learning and evaluate the importance of influencing factors	Secondary data analysis of prediction model	A tertiary hospital in Korea; 1409 nurses, 2011–2021 data	Characteristic: Machine Learning Models: decision tree, logistic regression, and random forest, use of Python libraries (Pandas and Scikit-learn). Level: predictive analytics	Supported proactive human resource management by predicting turnover and identifying key influencing factors.	High risk of bias (RoB)/unclear concern for applicability (PROBAST)

14	King et al. [45]	To demonstrate how nurse managers can use real-time workforce data to proactively identify and mitigate nurse burnout	Quality improvement	Inpatient units at a large pediatric hospital (Children's National); data used by unit nurse managers.	Characteristic: real-time data analytics; integrates the human resources information system (HRIS) and software-as-a-service (SaaS) dashboard (Laudio) Level: descriptive analytics	Supported real-time early detection, targeted interventions, and strategic engagement to mitigate nurse burnout and promote healthy work environments.	Met minimum quality criteria set (QI-MQCS)

15	Kohn et al. [46]	To evaluate the predictive utility of ward capacity strain metrics on clinical outcomes among survivors of acute respiratory failure	Retrospective cohort study	Five hospitals in the University of Pennsylvania Health System; 5052 visits across 43 wards	Characteristic: Machine Learning Models: least absolute shrinkage and selection operator (LASSO), auto-machine learning (AutoML), pipeline using decision trees, random forest, and other algorithms used to evaluate ward strain variables. Level: diagnostic analytics and predictive analytics	Provided data-informed insights to manage ward resources, optimize staffing, and improve patient discharge outcomes.	High-quality (JBI critical appraisal checklist for cohort studies)

16	Lindberg et al. [47]	To identify the most important factors in an inpatient fall risk prediction model using machine learning approaches applied to EHR and administrative data	Case–control study	University of Florida Health System; 814 patients (272 fallers and 542 nonfallers)	Characteristic: Machine Learning Models: single classification tree, bagging, random forest, and adaptive boosting; evaluation for sensitivity and specificity using the area under the receiver operating characteristic (AUROC). Level: diagnostic analytics and predictive analytics	Supported proactive fall prevention strategies and helped nurse managers prioritize high-risk patients and allocate resources effectively to improve patient safety and quality of care.	High-quality (JBI critical appraisal checklist for case–control studies)

17	Martinez et al. [48]	To implement and evaluate the advance alert monitor (AAM) program for early detection and intervention for patients at risk for in-hospital clinical deterioration	Quality improvement	Kaiser Permanente Northern California, 21 hospitals; analysis included 11,723 cases and controls	Characteristic: predictive analytics embedded in the EHR using 99 variables; supported by a virtual quality nurse consultant (VQNC) team that provided continuous monitoring and early warning alerts. Level: predictive analytics	Supported proactive management of high-risk patients and standardized workflows for rapid response teams.	Met minimum quality criteria set (QI-MQCS)

18	Moon [49]	To develop and evaluate the nursing resources management information system (NRMIS) in order to manage nurse resources efficiently	System development and evaluation study	National University Hospital in Korea; included 78 nurses managing 1122 patients across medical, surgical, and ICU units	Characteristic: NRMIS with components, such as a patient classification system (PCS), nursing staff scheduling system (NRS), and optimal nursing manpower estimating system (ONMES). Level: descriptive analytics	Provided objective data for flexible nurse resource allocation, enhanced efficiency and accuracy of, and supported informed decision-making on staffing needs.	Good practice (GEP-HI)

19	Sandow and Bowie [50]	To develop and implement a predictive logistics engine to support nurse managers in proactively scheduling and staffing based on projected patient volumes and evidence-based staffing standards	Quality improvement	St. Luke's health system (Idaho); eight hospitals and multiple departments (inpatient and emergency). Sample included nurse managers and scheduling teams.	Characteristic: Microsoft Power BI; historical patient volume data (36 months), staffing standards, and nondirect care time. Level: predictive analytics	Enabled data-driven decision-making for scheduling, hiring, paid time off (PTO) approval, and workforce planning; increased forecasting accuracy; and improved staff satisfaction and cost control.	Met minimum quality criteria set (QI-MQCS)

20	Xu et al. [51]	To predict nurse turnover using machine learning techniques with highly imbalanced data	Retrospective observational study	The 2018 U.S. National Sample Survey of Registered Nurses (NSSRN), 43,987 records	Characteristic: Machine Learning Models: random forest, extreme gradient boosting (XGBoost), and decision trees. Level: predictive analytics	Provided data-informed insights for early identification of turnover risks.	Excellent (STROBE tool)

21	Yan et al. [52]	To construct and validate machine learning algorithms for predicting chronic postsurgical pain (CPSP) among patients undergoing total knee arthroplasty	Prospective cohort study	Two hospitals in China; 320 patients in the modeling group and 150 patients in the validation group	Characteristic: Machine Learning Algorithms: random forest, support vector machine (SMV), K-nearest neighbors, and decision tree Level: diagnostic analytics and predictive analytics	Enabled nurse managers to improved quality of care by leveraging identification of patients at high risk for CPSP.	High-quality (JBI critical appraisal checklist for cohort studies)

**Table 2:** Quality appraisal summary (*n* = 21).

Quality appraisal tool	Study design	Total studies *n* (%)	Study no.
JBI	Cross-sectional	4 (19.05)	1, 4, 7, 9
	Case–control	2 (9.52)	11, 15
	RCT	1 (4.76)	12
	Cohort	2 (9.52)	14, 19

PROBAST	Prediction model	1 (4.76)	13

STROBE	Observational	3 (14.29)	2, 10, 18

GEP-HI	Health informatics	5 (23.81)	3, 5, 6, 8, 17

QI-MQCS	Quality improvement	3 (14.29)	14, 18, 19

**Table 3:** The impact of data analytics on nursing management roles.

Level of data analytics	Nursing management roles			
	Improving patient care quality	Strategic management	Nurse staffing and work engagement	Nursing management during health crises
Descriptive analytics	Delivered information to support clinical decision-making, enhance nursing workflows, and improve patient care quality (1)		Supported effective nurse staffing and promoted healthy work environments (2, 14, 18)	
Diagnostic analytics	Enhanced decision transparency and efficiency to optimize clinical workflows (3)	Provided insights into bed occupancy and ward-level data (5)	Identified workload-related factors that affect effectiveness of staff workload management (5)	
Predictive analytics	Supported proactive management on high-risk patient (17) and identified workload factors and allostatic load to optimize care delivery (4, 10, 11)		Improved nurse staffing patterns (10), identified nurse mental health's predictors (9), and predicted nursing staff turnover and burnout to inform proactive strategies (7, 13, 19, 20)	
Prescriptive analytics		Helped informed strategic and operational decisions in optimizing resource allocation to prepare for pandemic surges (6)	Optimized workforce planning and resource deployment (6), identified risk prediction, and recommended standardized protocols (12)	Guided resource allocation during pandemic surges and enhanced crisis response with real-time simulation tools (6)
Integrated analytics	Combined predictive and prescriptive analytics to streamline patient scheduling and reduce waiting times (8), and helped prioritize high-risk patients to improve patient safety and quality of care (16, 21)	Enabled proactive and informed decision-making through comprehensive models to manage ward resources (15)	Enabled data-informed insights to enhance workload balance and optimize nurse scheduling (8, 15)	

**Table 4:** Matrix of nurse managers' administrative decision-making process [14] and the impact of data analytics.

Administrative decision-making process	The impact of data analytics				
	Descriptive analytics	Diagnostic analytics	Predictive analytics	Prescriptive analytics	Integrated analytics
1. Defining the problem	Provided real-time information to help identify problems (1, 2, 14, 18)	Diagnosed root causes of operational challenges, such as workflows and staffing needs (3, 5)	Identified workload factors affecting near misses in nursing using big data (4) and anticipated staff turnover by identifying key factors (13)		Combined diagnostics with predictive analytics to comprehensively define problems (15, 16, 21)
2. Developing and ranking criteria		Highlighted critical factors for decision-making, such as ward-level constraints (5)	Prioritized risks and opportunities, such as predictors of nurse turnover or burnout (7, 9)		Integrated diagnostic and predictive insights to establish actionable criteria (15)
3. Collecting information	Summarized and delivered essential information for decision-makers (1, 2, 14, 18)	Provided insights of operational processes through decision logs developed from real-life dataset simulation and synthetic data (3)	Identified scenarios, including turnover risks and resource demands, to support proactive decision-making strategies (10, 19)		Combined data from multiple analytics levels to ensure comprehensive information collection (8, 12, 21)
4. Formulating and ranking solutions		Supported the development of solutions through clinical decision support system providing an algorithm using decision logs (3)	Enabled prioritization of high-risk patients and prediction of solution outcomes for clinical integration (11)	Provided optimal solutions applying queueing theory and microsimulation models to prepare for specific situation, such as pandemic surges (6)	Combined predictive and prescriptive capabilities to rank actionable solutions effectively (8, 12)
5. Jointly choosing the best solution			Predicted the effectiveness of proposed solutions, facilitating collaborative decision-making (17)		Combined insights across analytics levels to support collaborative decision-making (15)
6. Enacting and monitoring solutions		Tracked the effectiveness of implemented solutions, such as workload management (5)	Monitored ongoing impacts, enabling proactive adjustments to solutions, such as staff retention strategies (13)	Directly supported implementation through actionable recommendations and workflow improvements (6)	Enabled iterative monitoring and optimization across analytics levels (8, 12, 21)

## Data Availability

The data that support the findings of this study are available from the corresponding author upon reasonable request.
